# ‘The children of those who died of kuru are today still alive’

**DOI:** 10.1098/rstb.2008.4022

**Published:** 2008-11-27

**Authors:** Tiu Pekiyeva

**Affiliations:** Awarosa Village, c/o Papua New Guinea Institute of Medical ResearchPO Box 60, Goroka, EHP 441, Papua New Guinea

I was approximately 12 years old living in Agakamatasa village when Dr Carleton Gajdusek came and stayed with us in the village. I was selected by Carleton among other boys to help him take photographs of the village and the people. We toured other provinces and visited Lae, Madang, Rabaul, Hoskins, Chimbu and Mt Hagen in search of kuru disease but we found nothing. We settled down in South Fore and worked. When a field reporter informed us of a new kuru patient, I used to go out and collect samples and take them to Okapa to be sent to the United States.

I also worked with Jack Baker, who made life easier for us to go out into villages where kuru was claiming lives at an alarming rate and the people were anxious. The women and children were worst affected by the disease, leaving many orphans behind for family members to look after. Lately, we have not seen or heard of real kuru, and the children of those who died of kuru are today still alive in the villages. However, those of us who saw the worst of the kuru epidemic are still in fear of whether it will come back or not.

